# Carbon Nitride Gels: Synthesis, Modification, and Water Decontamination Applications

**DOI:** 10.3390/gels11090685

**Published:** 2025-08-27

**Authors:** Qinglan Tang, Zhen Zhang, Yuwei Pan, Michael K. H. Leung, Yizhen Zhang, Keda Chen

**Affiliations:** 1 College of Safety and Environmental Engineering, Shandong University of Science and Technology, Qingdao 266590, China; 2 Ability R&D Energy Research Centre, School of Energy and Environment, City University of Hong Kong, Hong Kong, China; mkh.leung@cityu.edu.hk; 3 College of Biology and the Environment, Nanjing Forestry University, Nanjing 210037, China

**Keywords:** carbon nitride, photocatalyst, hydrogel, aerogel, water decontamination

## Abstract

Graphitic carbon nitride (g-C_3_N_4_)-based materials hold significant promise for environmental remediation, particularly water purification, owing to their unique electronic structure, metal-free composition, and robust chemical stability. However, powdered g-C_3_N_4_ faces challenges such as particle aggregation, poor recyclability, and limited exposure of active sites. Structuring g-C_3_N_4_ into hydrogels or aerogels—three-dimensional porous networks offering high surface area, rapid mass transport, and tunable porosity—represents a transformative solution. This review comprehensively examines recent advances in g-C_3_N_4_-based gels, covering synthesis strategies such as crosslinking (physical/chemical), in situ polymerization, and the sol–gel and template method. Modification approaches including chemical composition and structural engineering are systematically categorized to elucidate their roles in optimizing catalytic activity, stability, and multifunctionality. Special emphasis is placed on environmental applications, including the removal of emerging contaminants and heavy metal ions, as well as solar-driven interfacial evaporation for desalination. Throughout, the critical interplay between gel structure/composition and performance is evaluated to establish design principles for next-generation materials. Finally, this review identifies current challenges regarding scalable synthesis, long-term stability, in-depth mechanistic understanding, and performance in complex real wastewater matrices. This work aims to provide valuable insights and guidance for advancing g-C_3_N_4_-based hydrogel and aerogel technologies in environmental applications.

## 1. Introduction

Water pollution has become a global environmental crisis due to the rapid discharge of industrial effluents, agricultural runoff, and emerging contaminants such as pharmaceuticals, heavy metals, and microplastics [1]. Conventional water treatment technologies (e.g., adsorption, coagulation, and biological degradation) often suffer from inefficiency, high energy consumption, or secondary pollution [2,3,4]. Advanced oxidation processes (AOPs), particularly photocatalysis, offer a sustainable solution by utilizing solar energy to degrade pollutants into harmless products [5]. However, the development of efficient, stable, and reusable photocatalytic materials remains a critical challenge.

Graphitic carbon nitride (g-C_3_N_4_), a metal-free polymeric semiconductor, has emerged as a promising photocatalyst due to its visible-light responsiveness (~2.7 eV bandgap), chemical stability, and low-cost synthesis from nitrogen-rich precursors [6]. It possesses a unique electronic structure and excellent chemical stability, with a layered and highly stable structure. However, its practical application is hindered by limited photocatalytic efficiency: (1) Fast charge recombination and insufficient active sites reduce quantum yield [7]. (2) Aggregation tendency: Bulk g-C_3_N_4_ tends to stack, reducing accessible surface area and reaction sites [8]. (3) Difficulty in recovery: Powdered g-C_3_N_4_ is hard to separate from treated water, causing secondary pollution [9].

Gel materials are a type of functional materials with unique three-dimensional (3D) network structures [10] and have shown extensive application potential in multiple fields due to their special physical and chemical properties [11]. The advantages of gel materials mainly lie in three aspects: 3D microporous structure, high specific surface area (300–1200 m^2^ g^−1^), and controllable physical and chemical properties [12,13,14]. These characteristics make them a research hotspot in materials science and engineering applications. The most distinctive feature of gel materials is their 3D microporous structure, which is composed of cross-linked polymer chains or nanoparticles and forms a continuous pore network. This structure not only endows gel materials with excellent flexibility and elasticity but also enables them to adsorb and store a large amount of liquid or gas molecules. For example, in the field of water treatment, this 3D porous structure of gel materials can effectively adsorb pollutants, such as heavy metal ions and organic dyes [15]. The high specific surface area of gel materials stems from their nanoscale pores and abundant internal interfaces, which significantly enhances the adsorption capacity, catalytic activity, and mass transfer efficiency of the materials [16].

g-C_3_N_4_-based gel materials, particularly those derived from graphitic g-C_3_N_4_, have emerged as a versatile class of materials for environmental applications due to their unique physicochemical properties. These gels, characterized by their high surface area, tunable porosity, and abundant nitrogen-containing functional groups, exhibit excellent adsorption capacity, photocatalytic activity, and chemical stability. In water treatment, g-C_3_N_4_ gels are employed for the removal of organic pollutants, heavy metals, and emerging contaminants through mechanisms such as adsorption, photocatalysis, and synergistic processes. Their nitrogen-rich framework facilitates strong interactions with pollutants, while their semiconductor properties enable efficient degradation of organic compounds under visible light [17]. A notable example is the work by Han et al. [18], who developed a g-C_3_N_4_-based hydrogel via in situ polymerization for the photocatalytic degradation of dyes in wastewater. Their study demonstrated that the hydrogel’s porous structure and high surface area significantly enhanced the adsorption and subsequent photodegradation of methylene blue (MB), achieving over 90% removal efficiency within 60 min under visible light irradiation.

Recent reviews have highlighted the potential of g-C_3_N_4_-based materials in environmental remediation. For instance, Huo et al. [19] reviewed the synthesis and modification of g-C_3_N_4_ nanomaterials for photocatalytic water purification, emphasizing the role of bandgap engineering and heterostructure design in enhancing photocatalytic efficiency. Their work focused on nanostructured g-C_3_N_4_ composites and their applications in degrading organic pollutants, providing insights into material optimization but not specifically addressing gel-based systems. Similarly, Cheng et al. [20] summarized the advances in g-C_3_N_4_-based photocatalysts for water treatment, focusing on the integration of g-C_3_N_4_ with metal oxides and carbon materials to improve charge separation and pollutant degradation. Combining g-C_3_N_4_ with gel materials is a common method to enhance the conductivity of the materials. The resulting materials can be used for catalytic water treatment [21,22]. More recently, Ruan et al. [23] advanced the discussion by examining hybrid g-C_3_N_4_/polymer systems, primarily focusing on membrane applications rather than gel networks. These reviews highlighted the importance of g-C_3_N_4_-based composite formation but omitted exploration of the unique properties of gel-based systems.

**Figure 1:**
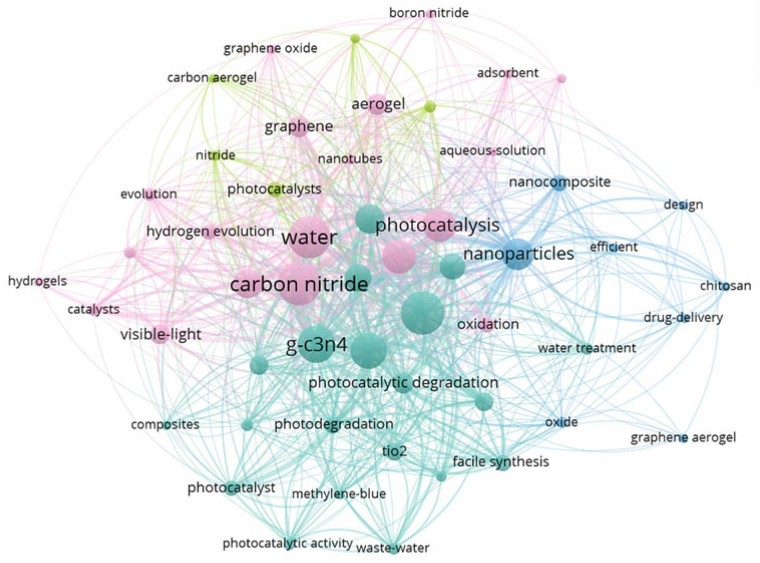
A co-occurrence network of research hotspots for g-C_3_N_4_-based gels in water treatment (1985–2025), constructed using VOSviewer (version 1.6.18).

Incorporating g-C_3_N_4_ into gel networks (such as hydrogels, aerogels, or cryogels), offers several notable advantages. These notable benefits include hierarchical porosity, in which macropores larger than 50 nm improve mass transfer and mesopores between 2 and 50 nm enhance pollutant adsorption [23]. The 3D framework also inhibits g-C_3_N_4_ aggregation, resulting in a high surface area with increased accessibility of active sites [24]. Furthermore, the functionality of the material can be tuned through surface modifications such as doping or constructing heterojunctions, which optimizes its electronic structure and strengthens the synergy between adsorption and photocatalytic performance [25]. Monolithic gels enable convenient separation and reuse, addressing the recovery challenge of powders [23]. Figure 1 illustrates evolving research trends and interdisciplinary themes in g-C_3_N_4_-based gels in water treatment, visualized through keyword network analysis.

This review systematically summarizes recent advances in g-C_3_N_4_-based gel materials for water treatment, focusing on precursor selection and synthesis strategies of hydrogels and aerogels. Modification approaches include elemental doping, surface functionalization, interfacial composition engineering, and hierarchical porosity engineering to optimize light absorption, charge separation, and pollutant affinity. Special emphasis is placed on environmental remediation applications, particularly the removal of heavy metal ions and emerging contaminants, alongside solar-driven interfacial evaporation for seawater desalination. By correlating material design with application performance, this review aims to guide the development of next-generation g-C_3_N_4_ gels for sustainable water purification.

## 2. Synthesis Strategies of Carbon Nitride-Based Gels

g-C_3_N_4_-based gel materials have attracted significant attention in water treatment due to their excellent photocatalytic properties, abundant functional groups, and tunable porous structures. The synthesis strategy plays a vital role in optimizing their structure and performance. This section systematically introduces the synthesis strategies of g-C_3_N_4_-based gels from three aspects: precursor selection, hydrogel synthesis methods, and aerogel synthesis methods.

### 2.1. Precursor Selection

The choice of precursors is critical in determining the structure, properties, and performance of g-C_3_N_4_-based gel materials. Common precursors can be categorized into synthetic and natural sources, each offering distinct advantages for tailored synthesis.

#### 2.1.1. Nitrogen-Rich Cyanamide-Based Precursors

Synthetic precursors such as cyanamide, dicyandiamide, and melamine are widely used due to their high nitrogen content and ability to form g-C_3_N_4_ structures through thermal polymerization. Cyanamide, a highly reactive monomer, facilitates the formation of highly ordered g-C_3_N_4_ networks under controlled heating (400–600 °C) [24]. Dicyandiamide, with its stable dimeric structure, is preferred for its ease of handling and ability to yield crystalline g-C_3_N_4_ with tunable porosity [25]. Melamine, a cost-effective and abundant precursor, is commonly employed to produce bulk g-C_3_N_4_, which can be further exfoliated or modified to form gel-like structures [26]. These precursors allow precise control over the chemical composition and degree of polymerization, enabling the design of gels with specific functionalities for water treatment applications.

#### 2.1.2. Natural Biomass Precursors

To enhance sustainability, natural biomass precursors such as chitosan, cellulose, and lignin have gained attention. These renewable materials serve as both carbon and nitrogen sources, reducing reliance on synthetic chemicals. For instance, chitosan, derived from crustacean shells, provides amino-rich groups that facilitate the formation of nitrogen-doped gel frameworks. Cellulose-based precursors contribute to the formation of porous structures, enhancing the mechanical stability of gels [27]. Lignin, a byproduct of the paper industry, can be thermally treated to yield g-C_3_N_4_-like materials with hierarchical porosity [28]. Biomass-derived gels are eco-friendly and cost-effective, aligning with the principles of green chemistry [29], though challenges remain in achieving consistent chemical compositions and scalable production. Recent work [30] also demonstrates the viability of industrial waste (e.g., glass powder) as low-cost precursors for functional materials.

### 2.2. Hydrogel Synthesis Methods

Hydrogels based on g-C_3_N_4_ are synthesized through various methods, leveraging physical or chemical interactions to form stable, water-swollen networks. These methods allow control over the gel’s mechanical properties, porosity, and functionality.

#### 2.2.1. Physical Crosslinking

**Figure 2:**
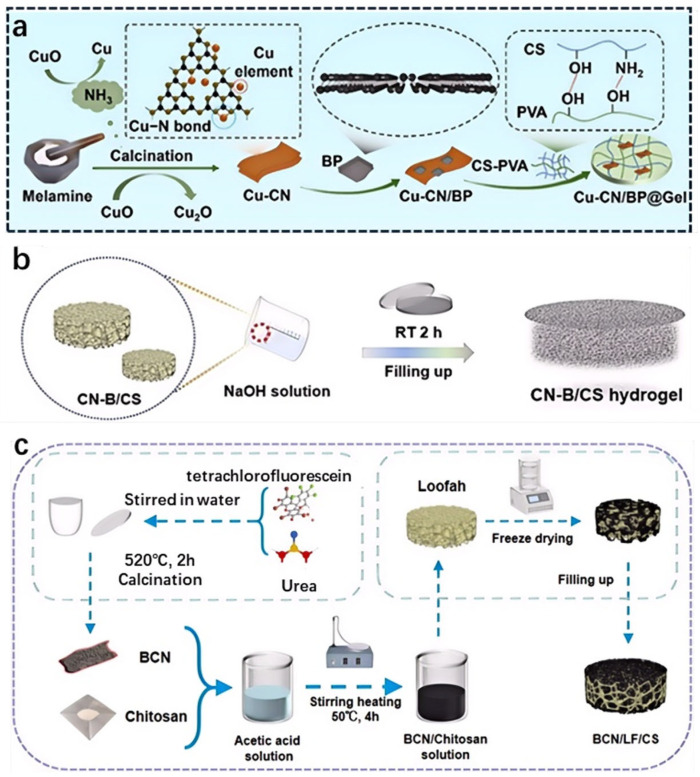
(**a**) Schematic illustration of the Cu-CN/BP@Gel hydrogel [33]. (**b**) Schematic illustration of the CN-B/CS hydrogel [36]. (**c**) Schematic illustration of the preparation of the BCN/LF/CS solar evaporator by the continuous process [39].

Physical crosslinking relies on non-covalent interactions, such as hydrogen bonding and van der Waals forces, to form reversible hydrogel networks. For g-C_3_N_4_-based hydrogels, precursors like melamine can be dispersed in aqueous media, where hydrogen bonds between nitrogen-rich functional groups (e.g., -NH_2_; =NH) stabilize the gel structure [31,32]. Additives such as polyols or ionic liquids can enhance these interactions, improving gel elasticity and swelling capacity [33]. Figure 2a illustrates the synthesis of a copper-g-C_3_N_4_ (Cu-CN)-based gel composite. The process begins with melamine and CuO, which undergo calcination to form Cu-CN. This is further processed with black phosphorus (BP) to create Cu-CN/BP. Subsequently, chitosan (CS) and polyvinyl alcohol (PVA) are incorporated through physical crosslinking, likely via hydrogen bonding and other intermolecular interactions, to form the final Cu-CN/BP@Gel composite. The physical crosslinking involves mixing CS and PVA with the Cu-CN/BP material, allowing the polymer chains to entangle and form a gel network without the need for chemical crosslinkers. Physical crosslinking is advantageous for its simplicity and reversibility, allowing easy modification of gel properties. However, these gels often exhibit lower mechanical strength, limiting their application in high-stress environments.

#### 2.2.2. Chemical Crosslinking

Chemical crosslinking involves the formation of covalent or ionic bonds to create robust hydrogel networks. Covalent crosslinking can be achieved by incorporating crosslinkers like glutaraldehyde or epichlorohydrin, which react with the amine groups of g-C_3_N_4_ precursors to form stable networks. Ionic crosslinking, often using divalent cations (e.g., Ca^2+^; Mg^2+^), introduces electrostatic interactions that enhance gel stability [34,35,36]. Figure 2b illustrates the formation process of the CN-B/CS hydrogel. It starts with CN-B/CS discs, which are treated with an NaOH solution. The solution is used to fill up the discs, followed by a reaction at room temperature for 2 h (RT 2 h), resulting in the formation of the CN-B/CS hydrogel. These methods produce hydrogels with superior mechanical strength and durability, making them suitable for repeated use in water treatment processes. However, the use of chemical crosslinkers may introduce toxicity, requiring careful selection of biocompatible agents.

#### 2.2.3. In Situ Polymerization

In situ polymerization, particularly free radical polymerization, enables the direct formation of g-C_3_N_4_-based hydrogels within a reaction medium. Monomers such as acrylamide or acrylic acid are copolymerized with g-C_3_N_4_ precursors in the presence of initiators (e.g., ammonium persulfate) and crosslinkers (e.g., N, N′-methylenebisacrylamide) [37,38,39]. Figure 2c demonstrates the preparation process of the BCN/LFCS. It begins with BCN and chitosan, which are stirred in water and an acetic acid solution containing urea and tetrachlorofluorescein, respectively. The mixture undergoes calcination at 520 °C for 2 h. Separately, loofah is subjected to freeze-drying. The resulting BCN/chitosan solution is then combined with the freeze-dried loofah, followed by filling up and further processing, ultimately yielding the BCN/LFCS hydrogel. This results in the formation of a robust nanocomposite hydrogel, where CNBs serve as reinforcing agents within the polymer network. This method allows precise control over the gel’s microstructure and functionalization, enabling the incorporation of active sites for pollutant adsorption or photocatalysis. The resulting hydrogels exhibit high water retention and tunable porosity, making them ideal for water treatment applications. However, the process requires careful optimization to avoid incomplete polymerization or phase separation.

### 2.3. Aerogel Synthesis Methods

g-C_3_N_4_-based aerogels are lightweight, highly porous materials synthesized through advanced techniques to achieve high surface area and structural stability. These methods focus on controlling the pore structure and drying process to preserve the architecture of gel.

#### 2.3.1. Sol–Gel Method with Supercritical Drying/Freeze-Drying

**Figure 3:**
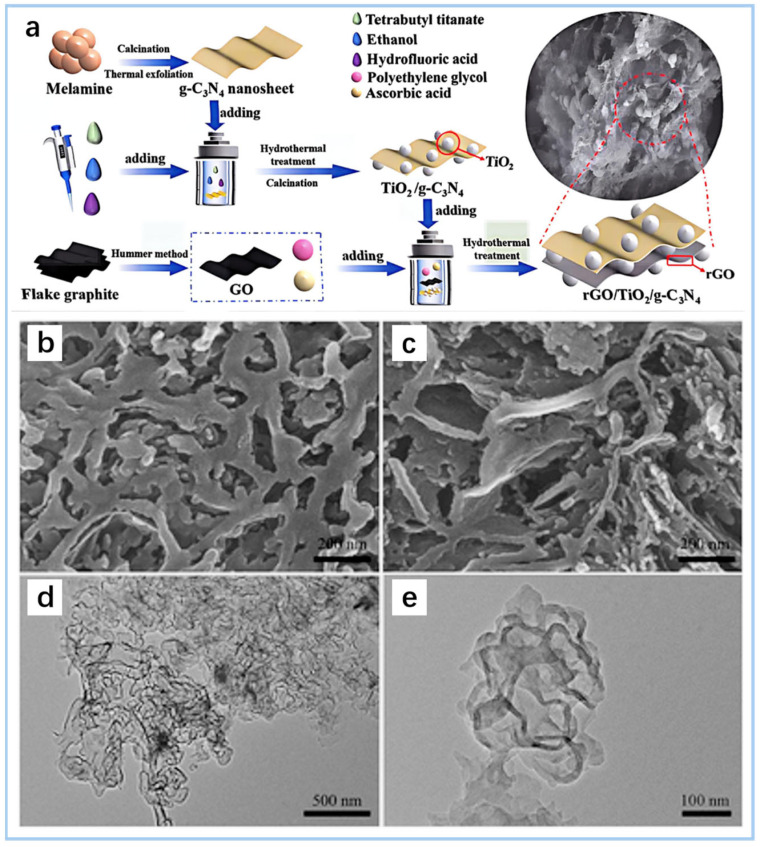
(**a**) Schematic illustration for the synthesis process of rGO/TiO_2_/g-C_3_N_4_ aerogels [42]. SEM images (**b**,**c**) from different areas and TEM images (**d**,**e**) with different magnifications for npg-CN-0.5c [44].

The sol–gel method, followed by supercritical drying or freeze-drying, is a common approach for synthesizing g-C_3_N_4_-based aerogels. In the sol–gel process, precursors are dispersed in a solvent to form a colloidal suspension, which transitions into a gel through controlled polymerization [40]. Supercritical drying, typically using CO_2_, removes the solvent while preserving the porous structure, yielding aerogels with high surface areas (up to 500 m^2^ g^−1^). Freeze-drying, an alternative method, involves freezing the gel and sublimating the ice under vacuum, producing aerogels with interconnected macropores. Sun et al. [41] reviewed the process of gelating carbon and nitrogen precursors through the sol–gel method, and then combining drying and heat treatment to prepare N-doped carbonized gel. rGO/TiO_2_/g-C_3_N_4_ composite aerogels were prepared by a sol–gel method according to the procedure and various steps (Figure 3a) [42]. Both techniques allow the formation of lightweight, mechanically stable aerogels suitable for adsorption and photocatalytic applications in water treatment. However, supercritical drying requires specialized equipment, while freeze-drying may lead to partial pore collapse if not optimized.

#### 2.3.2. Template Method (Soft/Hard Template for Pore Structure Control)

Template methods use soft or hard templates to precisely control the pore structure of g-C_3_N_4_-based aerogels. Soft templates, such as surfactants or block copolymers, self-assemble into micelles that guide the formation of ordered mesopores during gelation. Hard templates, such as silica nanoparticles or anodic aluminum oxide, provide rigid frameworks that are later removed (e.g., via etching) to yield hierarchical pore structures [43,44]. Deng et al. [44] used a soft template method (using Triton X-100) to prepare a patterned nitrogen-doped graphitic carbon nitride (npg-CN) with a nanoporous structure and studied the effects of template dosage and heat treatment temperature on the structure and photocatalytic performance. SEM and TEM images are shown in Figure 3b–e. These methods enable the design of aerogels with tailored pore sizes (2–50 nm) and high surface areas, enhancing their capacity for pollutant capture and mass transfer in water treatment. The template approach offers excellent control over morphology but requires additional steps for template removal, which can increase production costs and complexity.

These synthesis strategies enable precise control over the texture, surface chemistry, and functionality of CN-based gels, paving the way for their application in water purification, catalysis, and pollutant removal.

## 3. Modification of g-C_3_N_4_-Based Gel Composite Photocatalyst

### 3.1. Chemical Composition Tuning

Chemical composition tuning is one of the core strategies for optimizing the performance of C-N gel-based composite photocatalysts. By precisely introducing hetero-elements at the molecular or atomic scale, modifying surface functional groups, or regulating the chemical state of the interface, the inherent limitations of pure g-C_3_N_4_ gel in terms of light absorption range, carrier separation efficiency, and surface reactivity can be effectively overcome, making it more suitable for the demands of complex water treatment scenarios, such as wide-spectrum response, strong redox capacity, selective adsorption, and degradation of target pollutants [45]. The main strategies include element doping and surface functional group modification.

#### 3.1.1. Elemental Doping

**Figure 4:**
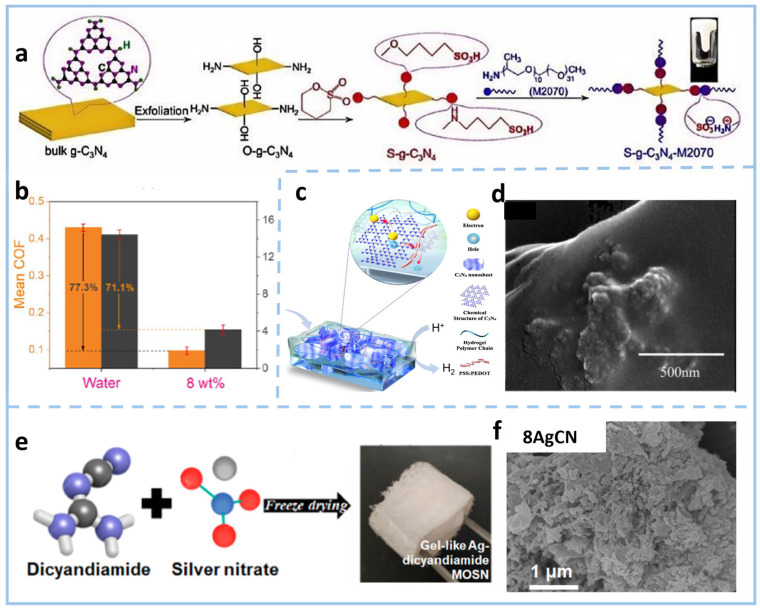
(**a**) Schematic diagram of the synthesis process of the S-g-C_3_N_4_-M2070 nanosheet hybrid. (**b**) Mean coefficient of friction and wear volume of water and S-g-C_3_N_4_-M2070 nanosheet hybrid dispersion (8 wt%) with load in 120 min [49]. (**c**) Suspension-mimicking hydrogel-based film composed of B-g-C_3_N_4_ nanosheets with charge transfer pathways. (**d**) SEM image of B-g-C_3_N_4_ nanosheets encapsulated by hydrogel matrix [50]. (**e**) Ag-contained g-C_3_N_4_ derived from the gel-like Ag-dicyandiamide metal−organic supramolecular network. (**f**) SEM image of 8AgCN [52].

Element doping can directly modify the electronic structure, charge distribution, and surface properties of materials by introducing heteroatoms into the g-C_3_N_4_ framework or anchoring them on its surface and in its pores, thereby optimizing their photocatalytic and adsorption performance. This strategy is mainly divided into non-metallic doping and metallic doping. In terms of non-metallic doping [46]. S/O doping is mostly introduced by substituting N atoms [47] or regulating nitrogen-rich defects in g-C_3_N_4_ [48], which can form new surface states on the material surface or regulate the energy band structure. It can not only improve the oxidation capacity but also enhance the hydrophilicity of the material and its affinity for pollutants. B/P doping is usually achieved by substituting C or N atoms in the g-C_3_N_4_ framework. This can change the electron cloud density of the framework, effectively narrow the bandgap, enhance the material’s ability to absorb visible light, and promote the separation efficiency of photogenerated carriers. It particularly has a significant effect on improving the catalytic activity for the reduction in oxygen-containing anions (such as nitrate). Zhao et al. [49] constructed a gel-like S-g-C_3_N_4_-M2070 nanosheet hybrid from the bulk g-C_3_N_4_ through the steps of Hummer’s method, sulfonation, and acid-base neutralization. The material features a core–shell structure, with g-C_3_N_4_ nanosheets as the core and organic modifiers as the shell component. This S-doped CN gel exhibits excellent dispersion stability in water (Figure 4a). When utilized as a water-based additive, adding 8 wt% can reduce the friction coefficient and wear volume of water by 73.7% and 72.3%, respectively (Figure 4b). Additionally, it shows outstanding load-bearing capacity and durability in long-term tests under high load conditions. In another study, a boron-doped graphite-like carbon nitride (B-g-C_3_N_4_) nanosheet hydrogel photoelectrode has been prepared at a heating rate of 3 °C min^−1^ to 600 °C and kept for 4 h under an argon atmosphere [50] (Figure 4c). Temperature and atmosphere are critical factors in the synthesis of g-C_3_N_4_ gels, influencing structural integrity, electronic properties, and functionality. By introducing shallow trap states on the surface through boron doping, efficient electron capture and charge separation were achieved. The surface was rough (Figure 4d), but the hydrogel matrix provided a suspension-like environment for the nanosheets, reducing agglomeration and shortening the charge migration distance, while allowing free diffusion of water molecules and gases. This synergy resulted in an increase in the photocurrent density by an order of magnitude compared to the ordinary g-C_3_N_4_ photoelectrode at 0.3 V vs. RHE.

There are more diverse implementation paths for metal doping, including coordination-assisted gelation (using metal ions as cross-linking agents or comonomers to participate in the construction of gel networks), post-ion exchange/adsorption methods (fixing metal ions by utilizing the adsorption performance of gel networks after they adsorb metal ions), and photo/chemical deposition methods [51]. These methods can regulate the electronic structure of materials through the introduction of metal ions, further optimizing their photocatalytic activity and stability. By introducing impurity atoms such as Ag, the electrical conductivity and light response of it can be significantly improved. A silver-containing dicyandiamide metal–organic supramolecular network (MOSN) gel [52] has been used as the precursor, and Ag-doped g-C_3_N_4_ (xAgCN) was prepared through freeze-drying and calcination at 550 °C for 4 h at a ramp rate of 2.5 °C min^−1^ (Figure 4e,f). The introduction of silver formed a metal–semiconductor interface, promoting charge separation, enhancing visible light absorption, and inhibiting charge recombination. Among them, 8AgCN exhibited the best performance. 8AgCN obtained after heat treatment has a specific surface area which increases from 8.2 m^2^ g^−1^ to 10.8 m^2^ g^−1^, exhibiting Type IV isotherms with hysteresis loops, indicating a mesoporous structure. The smaller sheet-like structure of 8AgCN is consistent with its larger specific surface area, which can provide more active sites for photocatalytic reactions.

#### 3.1.2. Surface Functional Group Modification

The surface of the carbon–nitrogen gel was chemically treated to modify its surface groups without altering the core framework structure. The surface groups of the g-C_3_N_4_ gel were modified through chemical means including oxidation and amination, which introduce polar functional groups (-COOH, -CN, etc.) without disrupting the basic framework of g-C_3_N_4_ to regulate it, achieving the synergistic enhancement of adsorption–photocatalysis. This significantly alters the surface energy and hydrophilicity/hydrophobicity [53]. The oxygen-containing groups (such as -COOH) enhance the interaction with water molecules through hydrogen bonding, improving the dispersion and stability of the material in water. This is in synergy with the three-dimensional network structure of the gel, expanding the contact interface with pollutants [54]. The N-containing or S-containing groups such as -CN capture specific charged pollutants through charge interaction or the chelation effect, demonstrating the logic of structural design and function-oriented regulation [55].

**Figure 5:**
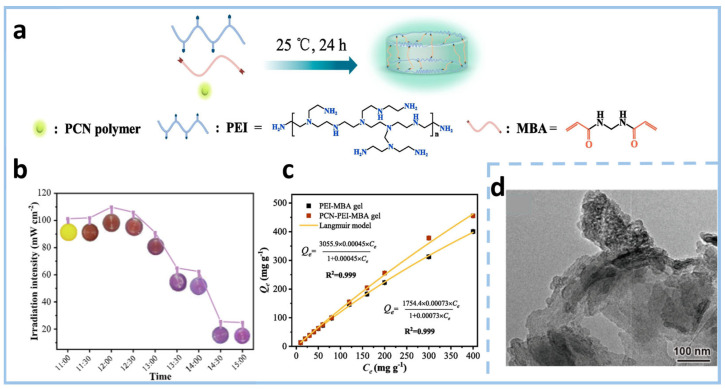
(**a**) Schematic illustration of the fabrication process for PCN-PEI-MBA hydrogel. (**b**) Variation in solar irradiation intensity over time and the color evolution of PCN-PEI-MBA hydrogel following Cr(VI) (10 mg L^−1^) adsorption and subsequent sunlight irradiation. (**c**) Adsorption isotherm of PCN-PEI-MBA and PEI-MBA gels for Cr(VI) [57]. (**d**) Transmission electron microscopy (TEM) micrograph of CCN [56].

Researchers regulated the surface functional groups by preparing phenyl-modified carbon nitride (PCN) polymers. They used phenylguanidine carbonate as a precursor and carried out thermal polymerization (Figure 5a). Then, they combined these polymers with polyethyleneimine- N, N′-Methylenebisacrylamide (PEI-MBA) gel to obtain phenyl-functionalized CN gel (PCN-PEI-MBA gel) at 350 °C with a rate of 2.3 °C min^−1^ with an N_2_ atmosphere. This gel has the ability to detect Cr(VI) through both fluorescence and colorimetry dual-mode detection (Figure 5b). Its saturated adsorption capacity for Cr(VI) is as high as 3055.9 mg g^−1^, far exceeding existing adsorbents (Figure 5c). Under visible light irradiation, it can photocatalytically reduce the adsorbed Cr(VI) to Cr(III), and the reduction process can be monitored in real time through color changes. Finally, Cr(III) can be recovered as Cr_2_O_3_ through calcination. In addition to this, polymeric carbon nitride (PCN) [56] has been synthesized through thermal polymerization as the precursor. Through a two-step process involving the melt-salt method and self-assembly, researchers prepared a crystalline carbon nitride (CCN) self-supporting aerogel functionalized with cyanide (-CN) functional groups. This cyanide-doped CN gel has a three-dimensional interconnected porous structure, which can enhance the mass transfer efficiency of reactants and products and improve the light absorption capacity through multiple photon reflections (Figure 5d).

### 3.2. Structural Engineering

Constructing heterojunctions or designing multi-level pore systems to optimize the charge separation efficiency and mass transfer capacity of carbon–nitrogen gels is a key strategy to overcome their inherent limitations. The specific gel morphology, such as interconnected 3D networks, provides abundant interfaces for heterojunction formation (e.g., g-C_3_N_4_/MOFs; g-C_3_N_4_/rGO), where the tailored pore structure (size and distribution) regulates the contact area with reactants. This not only strengthens surface adsorption of pollutants via capillary action and specific surface interactions but also forms an internal electric field through interface band engineering, significantly promoting the separation of photogenerated charges by shortening electron transfer paths [58]. The multi-level pore design (mesopore–macropore synergy) enhances pollutant enrichment and diffusion by optimizing the mass transfer path [59]. These two strategies jointly address the bottleneck problems of high charge recombination rate and poor accessibility of active sites faced by carbon–nitrogen gels from the perspectives of electron transfer and material transport, providing a charge separation–mass transfer enhancement dual solution for water treatment applications [60].

#### 3.2.1. Interfacial Composition Engineering in Heterojunctions

The performance of heterojunctions is highly dependent on the chemical bonding at the interface, the distribution of elements, and the defect states. Precise control of the interface composition is crucial for constructing efficient charge transfer channels, with the focus of regulation concentrated on interface bonding, defects and chemical states, and element inter-diffusion [61]. By forming strong interactions such as covalent bonds and coordination bonds between the -NH_2_/-NH groups on the g-C_3_N_4_ gel surface and the active groups of other materials, close contact and electronic coupling can be achieved; interface defects or changes in element valence can serve as charge transfer bridges or active sites; the transition layer formed by element inter-diffusion can optimize band matching. The regulation methods include selecting components with complementary surface chemical properties, in situ growth of second phases using the gel network, surface functionalization pretreatment, controlling the composite conditions, etc. [17]. This is of great significance for water treatment, as the tightly bonded interface is the basis of efficient heterojunctions, which can retain strong redox capabilities and is suitable for scenarios where both oxidation and degradation of organic substances and reduction of heavy metals/nitrates are required [62]. Moreover, the active sites at the interface can directly participate in the adsorption and activation of pollutants.

**Figure 6:**
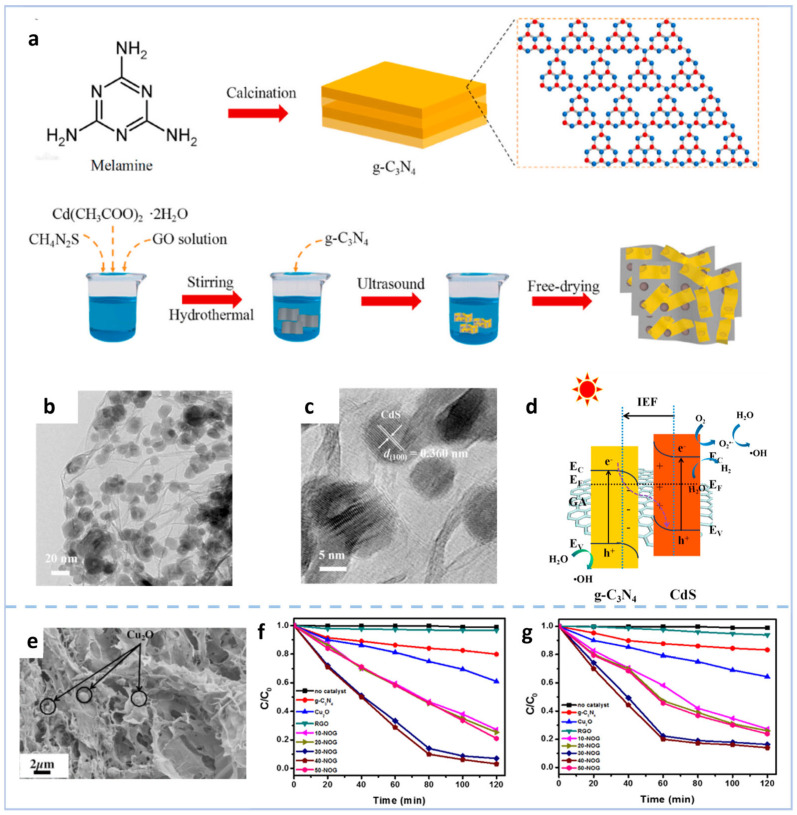
(**a**) Schematic diagram for synthesizing the CdS-g-C_3_N_4_-GA heterojunction. (**b**) TEM image and (**c**) HRTEM image of CdS-g-C_3_N_4_-GA. (**d**) Schematic illustration of CdS/g-C_3_N_4_ interfacial interaction and the S-scheme charge transfer mechanism in CdS-g-C_3_N_4_-GA composite under visible light [63]. (**e**) SEM images of 40-NOG aerogel. Photocatalytic activity of different samples’ photodegradation of (**f**) MB and (**g**) methyl orange [64].

For example, Liu et al. [63] employed an ultrasonic strategy to construct a CdS-g-C_3_N_4_-graphene aerogel (GA) ternary S-type heterojunction (Figure 6a), in which CdS nanoparticles and g-C_3_N_4_ sheets were uniformly dispersed within the three-dimensional porous structure of GA (Figure 6b,c). Compared with CdS-GA, the most abundant pore size of CdS-g-C_3_N_4_-GA shifted from 5.5 nm to 4.1 nm, which was attributed to the occupation of partial GA pores by g-C_3_N_4_ sheets. The specific surface area and pore volume of CdS-g-C_3_N_4_-GA were 152.9 m^2^ g^−1^ and 0.278 cm^3^ g^−1^, respectively, lower than those of CdS-GA. This reduction was caused by the introduction of g-C_3_N_4_, which itself possesses a low specific surface area (9.9 m^2^ g^−1^) and small pore volume (0.059 cm^3^ g^−1^). This heterojunction utilizes GA as an electron transport platform, allowing electrons to transfer from CdS through GA to g-C_3_N_4_, forming an intrinsic electric field that promotes the separation of photogenerated carriers. Under visible light, the S-type charge transfer mechanism retains strong redox capabilities (Figure 6d). It not only achieves a degradation efficiency of 91.4% for Rhodamine B, which is 40 times that of pure g-C_3_N_4_ and 11 times that of pure CdS, but also exhibits efficient hydrogen production performance (86.38 μmol h^−1^ g^−1^), with good cycling stability. Du et al. [64] prepared a ternary composite 3D aerogel of Cu_2_O/g-C_3_N_4_/RGO through self-assembly. In this composite, Cu_2_O forms a heterojunction with g-C_3_N_4_ and is loaded on the reduced graphene oxide sheets. This heterojunction structure expands the visible light absorption range and inhibits the recombination of photogenerated electron–hole pairs, and the aerogel with 40 wt% Cu_2_O content performs the best (Figure 6e). The degradation efficiency of MB and methyl orange is 96% and 83% within 80 min, respectively (Figure 6f,g). The introduction of RGO as an electron acceptor and bridge further promotes charge separation and transfer, improving the photocatalytic performance.

#### 3.2.2. Hierarchical Porosity Engineering

Hierarchical porosity engineering involves the elaborate design of g-C_3_N_4_-based gels with interconnected pores of varying sizes (micropores, mesopores, and macropores) to enhance mass transport and accessibility to active sites. Micropores boost surface adsorption via increased specific surface area and strong van der Waals interactions with pollutants, while mesopores and macropores facilitate rapid diffusion of reactants and products, ensuring efficient utilization of photogenerated charges [65,66]. This structural optimization is crucial for improving photocatalytic efficiency in water treatment applications, where rapid pollutant diffusion and effective light utilization are essential. The primary approaches include template-assisted methods, freeze-drying/solvent displacement, and in situ polymerization-etching. Among these, template-assisted strategies [67] utilize either hard templates (e.g., SiO_2_; MOFs) or soft templates (e.g., surfactants) to fabricate well-defined pore architectures. Freeze-drying (cryogelation) [68] employs ice-templating to induce aligned macroporous channels while retaining mesoporosity within the gel matrix. Additionally, self-assembly [69] processes enable the controlled aggregation of g-C_3_N_4_ nanosheets, leading to the formation of three-dimensional porous networks that further optimize mass transfer and light-harvesting capabilities.

**Figure 7:**
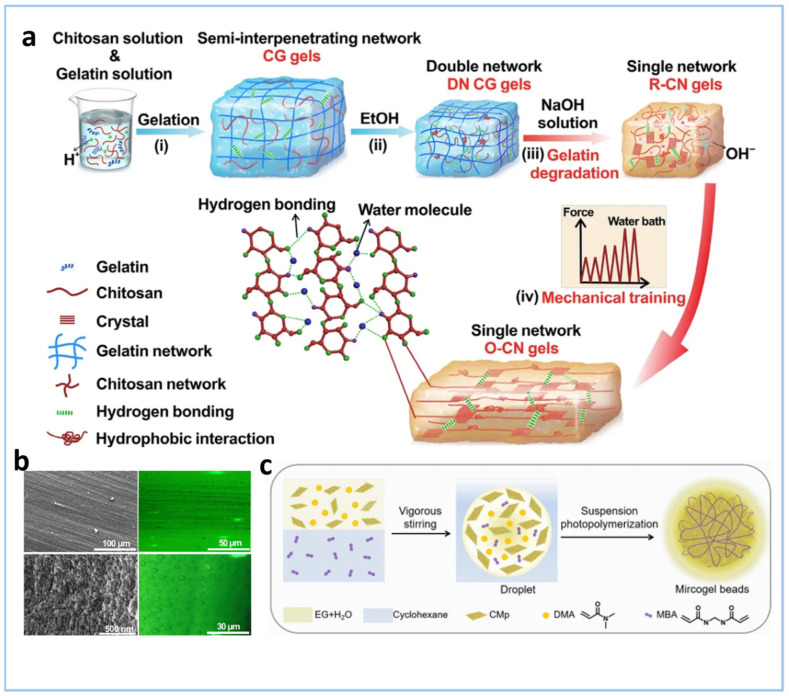
(**a**) Scheme of the preparation of crystal-mediated oriented chitosan nanofibrillar hydrogels (O-CN gels). Chitosan and gelatin solution were mixed to form semi-interpenetrating chitosan–gelatin gels (CG gels) at 4 °C due to the hydrogen bonding between gelatins. The chitosan chains dispersed into the gelatin network. (**b**) SEM (**left**) and LCSM (**right**) images of the surface and cross section of the O-CN gels [70]. (**c**) Overall synthetic procedure for PDMA/g-C_3_N_4_ MGB via Inverse Suspension Photopolymerization (DMA = N, N-Dimethylacrylamide; MBA = N,N′-Methylenebis(acrylamide); EG = Ethylene Glycol; CMp = Phenyl-Modified g-C_3_N_4_) [71].

Researchers have proposed a novel collaborative strategy for the preparation of O-CN gel through protein templating and mechanical training (Figure 7a) [70]. Firstly, chitosan and gelatin are mixed to form a semi-interpenetrating network chitosan–gelatin hydrogel (CG gel). Then, after ethanol treatment and soaking in sodium hydroxide solution, gelatin is degraded to form a crystal-mediated random chitosan nanofiber gel (R-CN gel). Finally, mechanical training is applied to the R-CN gel to induce an oriented nanofiber structure, resulting in the O-CN gel (Figure 7b). Du et al. [71] prepared PDMA/g-C_3_N_4_ MGBs large gel beads using a reversed suspension photopolymerization strategy (Figure 7c). The ternary heterojunction CdS-g-C_3_N_4_-GA achieved a specific surface area of 152.9 m^2^ g^−1^ and a pore volume of 0.278 cm^3^ g^−1^. Its multi-level pore structure, ranging from micropores to macropores, was primarily formed by adjusting both the size of the gel beads (30 μm–4 mm) and their internal pore architecture. This design enabled the gel beads to have controllable swelling properties (swelling ratios of 1.1–3.5). The smaller-sized gel beads, due to their higher porosity and specific surface area, enhanced their contact with pollutants, demonstrating excellent activity in rhodamine B degradation (97% degradation within 20 min) and exhibited good cycling stability.

## 4. Application of Carbon Nitride-Based Gel in Wastewater Purification

The escalating global challenge of water pollution, driven by industrial discharge, agricultural runoff, and domestic waste, demands innovative and sustainable solutions for wastewater treatment. Among emerging materials, carbon nitride-based gels (g-C_3_N_4_ gels) have emerged as versatile and promising candidates, leveraging their unique structural and chemical properties to address diverse contaminants in aqueous environments. The performance enhancement mechanism of carbon nitride gel-based composite photocatalysts focuses on the improvement of photocatalytic activity and the optimization of mechanical strength [72]. Through precise control of electronic behavior and fine design of microstructure, functional synergy is achieved, thereby breaking through the bottlenecks of traditional carbon nitride materials in practical applications. In terms of enhancing the photocatalytic activity, the core lies in addressing the key issues of narrow light absorption range and high charge recombination rate through the regulation of electronic structure and optimization of carrier dynamics [73]. In practical applications, carbon nitride gels have shown promise in treating industrial wastewater, decentralized water purification, and even natural water remediation. This section systematically introduces photocatalytic-based advanced oxidation processes (AOPs) for contaminant removal, as well as interfacial evaporation combined with photocatalytic pollutant degradation.

### 4.1. Photocatalytic AOPs for Contaminant Removal

#### 4.1.1. Organic Pollutant Removal

**Figure 8:**
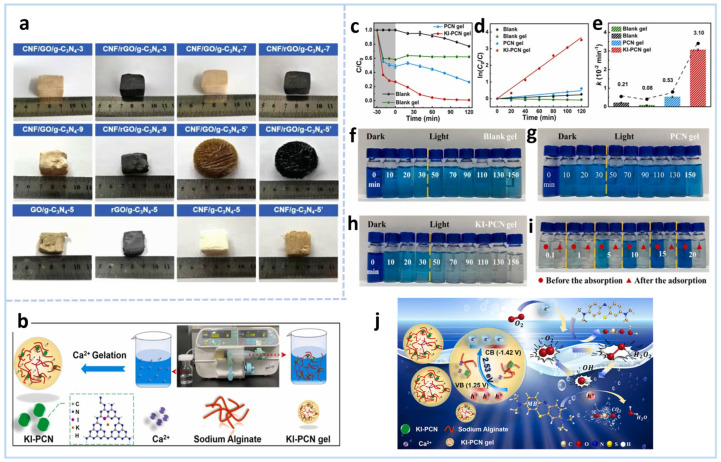
(**a**) Images of CNF/rGO/g-C_3_N_4_, rGO/g-C_3_N_4_, and CNF/g-C_3_N_4_ aerogels fabricated via bidirectional and conventional freeze-casting methods [74]. (**b**) Schematic illustration of KI-PCN hydrogel preparation. (**c**) Photocatalytic degradation curves and (**d**) corresponding pseudo-first-order kinetic fitting; (**e**) the apparent rate constant k and (**f**–**h**) time-dependent images of MB solution degradation over the reaction process. (**i**) The i photographs of MB solution before and after continuous adsorption using KI-PCN hydrogel. (**j**) Schematic of possible photocatalytic mechanism for MB degradation [77].

g-C_3_N_4_ gels excel in photocatalytic water treatment due to their 3D porous architecture, which provides a high surface area for enhanced pollutant adsorption and light harvesting. This structure facilitates efficient charge separation and transport, significantly suppressing electron–hole recombination while promoting the generation of reactive oxygen species. Consequently, their integrated macroscopic structure and tunable porosity make them promising candidates for industrial wastewater remediation, groundwater purification, and next-generation portable reactors targeting emerging contaminants. In a study, 3D compressible CNF/rGO/g-C_3_N_4_ aerogels were prepared via a simple bidirectional freeze-drying and thermal reduction process [74]. As depicted in Figure 8a, the rGO/g-C_3_N_4_-5 aerogel lacking CNFs underwent structural collapse and failed to retain its well-defined cubic morphology. Notably, CNFs not only inhibit the restacking of GO/g-C_3_N_4_ during the freezing process but also act as nanoskeletal supports to enhance the mechanical robustness of GO/g-C_3_N_4_ or pure g-C_3_N_4_ aerogels. Additionally, the disordered CNF/rGO/g-C_3_N_4_-5′ aerogel (prepared via conventional freeze-casting) was also characterized, revealing a randomly ordered pore architecture as evidenced by the morphological observations. The CNFs/rGO/g-C_3_N_4_-5 aerogel exhibited a specific surface area of 32.85 m^2^ g^−1^. Increasing g-C_3_N_4_ content elevated this value to 66.63 m^2^ g^−1^, exceeding literature reports (36.15 m^2^ g^−1^ and 20.8 m^2^ g^−1^) [75]. This enhanced surface area facilitates adsorption and photocatalytic kinetics by expanding active sites and accelerating mass transfer [76]. By adjusting the mass ratio of g-C_3_N_4_, the photocatalytic degradation performance of CNF/rGO/g-C_3_N_4_ aerogels for RhB and H_2_ production were evaluated. Under visible light irradiation from a 300 W Xe lamp equipped with a 420 nm cutoff filter, the RhB degradation efficiency reached 87.3%. The reactive oxygen species, including holes (h^+^), superoxide anion radicals (·O_2_^−^), and hydroxyl radicals (·OH), exhibit potent oxidizing capabilities, thereby driving the oxidation of organic pollutants.

A novel carbon nitride-based self-cleaning hydrogel photocatalyst—KI-PCN gel, comprising potassium-iodine co-doped carbon nitride confined within alginate matrices—was successfully constructed via a facile synthetic approach [77]. Beyond its exceptional photocatalytic performance, this material distinguishes itself with a remarkable self-cleaning capability, a critical feature for sustainable and recyclable water treatment. The preparation process is shown in Figure 8b. Notably, the blank gel sample was prepared using the same protocol as KI-PCN and PCN gels, with the sole distinction being the absence of catalyst addition. The KI-PCN gel exhibited a higher surface area (139.41 m^2^ g^−1^) than both the pristine (84.37 m^2^ g^−1^) and PCN gels (95.64 m^2^ g^−1^). This structural advantage further enabled superior synergistic adsorption–photocatalytic degradation of high-concentration methylene blue (HMB). Compared to limited self-degradation (24%) and adsorption-enhanced removal (32%) of 50 ppm MB by the blank gel, KI-PCN gel achieved near-complete degradation (99%) under visible light. Its reaction rate constant (0.0310 cm^−1^) surpassed the blank (0.0008 cm^−1^) and PCN gel (0.0053 cm^−1^), respectively (Figure 8c–e), primarily attributed to enhanced charge separation and active sites. Furthermore, the visual evolution of MB color during the degradation process is vividly depicted in Figure 8f–i. Strikingly, MB solutions treated with KI-PCN gel turned nearly colorless, while the color changes in the blank gel and PCN gel systems remained subtle—an observation consistent with their comparative degradation efficiencies.

Figure 8j illustrates the possible photocatalytic mechanism of KI-PCN gel for enhanced MB degradation. The gel structure significantly enhances photocatalytic performance through three key mechanisms: (1) the 3D porous network immobilizes and uniformly disperses photocatalysts like KI-PCN, preventing nanoparticle aggregation while maximizing active site exposure and pollutant accessibility; (2) co-doping with potassium and iodine (KI-PCN) narrows the bandgap for broader visible-light absorption, while iodine sites effectively trap electrons to suppress charge recombination; (3) functional groups (-NH_2_/-OH) on the gel matrix selectively adsorb and concentrate pollutants near catalytic sites, creating a synergistic adsorption–photocatalysis system that shortens degradation pathways and boosts efficiency. This integrated design overcomes critical limitations of powder catalysts in stability, light utilization, and recyclability. Hence, the 3D gel network effectively inhibits semiconductor particle aggregation through steric hindrance, while optimized crosslinking and interfacial chemical bonding establish directional charge transfer pathways. Furthermore, the precisely tailored pore structure (including pore size distribution and interconnectivity) significantly shortens carrier migration distances and minimizes charge recombination by reducing trapping sites. These structural advantages synergistically enhance charge separation efficiency, thereby optimizing the photocatalytic mechanism by promoting the generation and utilization of active species (e.g., ·OH and ·O_2_^−^) [78].

**Figure 9:**
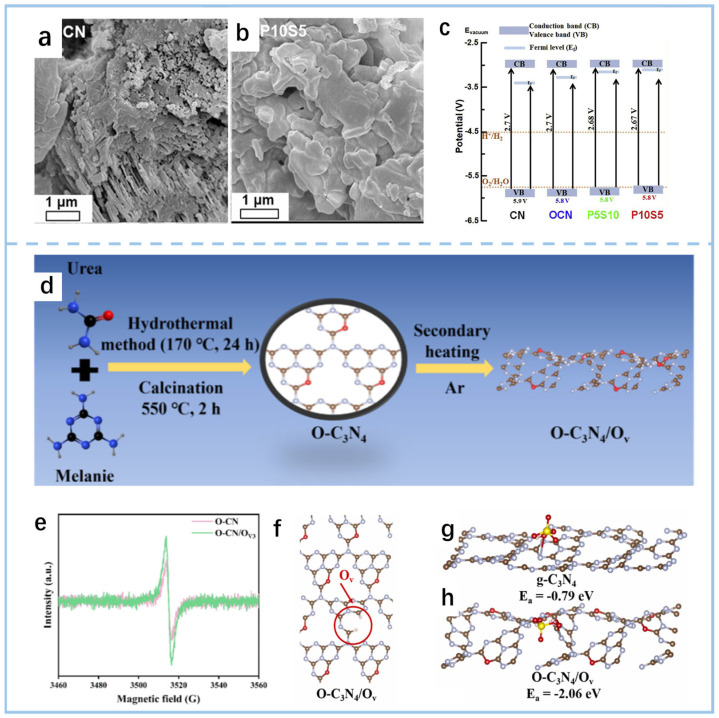
SEM images of (**a**) CN and (**b**) P10S5. (**c**) Band position diagrams of CN, OCN, P5S10, and P10S5 [79]. (**d**) The synthesis route of the catalyst. (**e**) ESR spectra of O-C_3_N_4_/Ov and O-CN. (**f**) O-C_3_N_4_/Ov model. (**g**,**h**) Adsorption energy of O-C_3_N_4_/Ov and g-C_3_N_4_ for PMS [80].

An enhancement in photocatalytic performance was achieved by preparing g-C_3_N_4_ doped with P, S, and O and subsequently forming a hydrogel [79] (Figure 9a,b). The bandgap modulation mechanism (Figure 9c) was mainly realized through element doping: P replaced the C atoms in g-C_3_N_4_ to form P-N bonds, while S and O replaced the N atoms to form C-S and C-O bonds. The valence band was raised slightly, while impurity levels were introduced to narrow the bandgap (2.67–2.70 eV). Theoretical calculations revealed that P doping induces charge transfer, localizing photogenerated electrons at P sites; S and O doping facilitate charge transfer between heptazine rings, and their synergistic effect enhances charge carrier separation efficiency, leading to significantly improved degradation performance and cycling stability of the co-doped g-C_3_N_4_ hydrogel under simulated sunlight. Additionally, some studies have integrated PMS (peroxymonosulfate) catalysis with photocatalysis, a synergistic strategy that has demonstrated significant potential in water treatment applications. Zhang et al. [80] achieved an enhancement in photocatalytic performance by preparing tubular g-C_3_N_4_(O-CN/O_v_) with oxygen doping and oxygen vacancies (O_v_) and forming recyclable hydrogel spheres and sponge sheets (Figure 9d). The electron trap inhibition mechanism mainly relies on the synergistic effect of oxygen doping and oxygen vacancies: oxygen doping forms C-O bonds, while oxygen vacancies (Figure 9e) are introduced through secondary calcination. Together, they optimize the electronic structure and reduce the recombination of photogenerated electron–hole pairs. Experimental and DFT calculations (Figure 9f–h) show that oxygen vacancies can capture photogenerated electrons, prolong carrier lifetime, and simultaneously reduce the adsorption energy and activation energy barrier of PMS, promoting the generation of active species (·O_2_^−^, h^+^, etc.); oxygen doping enhances electron transport ability and further inhibits carrier recombination.

#### 4.1.2. Removal of Metal Ions from Water

g-C_3_N_4_ gels exhibit a high specific surface area and hierarchical porous structures (comprising micropores, mesopores, and macropores), which maximize the accessibility of metal ions to active sites, thereby significantly enhancing their adsorption capacity. Furthermore, their surface chemistry is highly customizable: functional groups (e.g., -NH_2_, -OH, and -COOH) or heteroatom doping (e.g., S, P) can be introduced via simple post-synthetic modifications. This enables selective binding of target metal ions (such as Pb^2+^, Cd^2+^, and Cu^2+^) through electrostatic attraction, coordination, or redox interactions. Specifically, a novel adsorbent was fabricated by modifying chitosan: vanillin-crosslinked chitosan was integrated with thiourea-based g-C_3_N_4_ to form a gel matrix [81]. Adsorption experiments targeting Cu(II) and Cr(VI) ions revealed pseudo-second-order kinetics and Langmuir isotherm behavior, with endothermic, spontaneous thermodynamics. Desorption studies confirmed its regenerability, enabling repeated use for adsorbate recovery.

**Figure 10:**
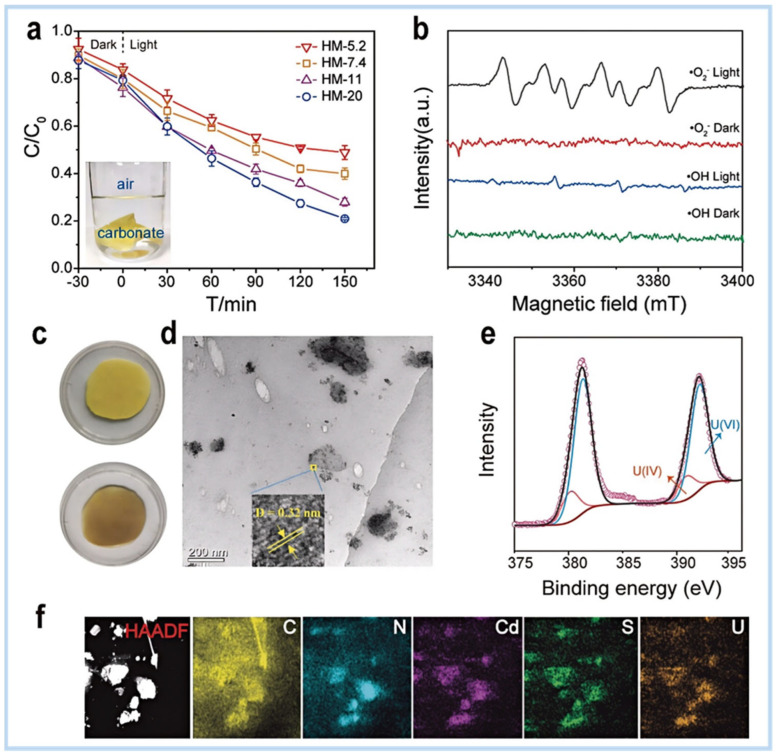
Multifaceted characterization of photocatalytic U(VI) reduction on hydrogel membranes: (**a**) reaction kinetics in air with 2.0 mM HCO_3_^−^; (**b**) DMPO spin-trapping EPR spectra of HM-20 under dark/visible light; (**c**) optical images of hydrogel before/after U(VI) reduction; (**d**) TEM micrographs of post-reaction membranes; (**e**) U 4f XPS spectra of reduction products; (**f**) elemental mapping of reacted membranes [83].

Terrestrial uranium ores, though critical for current nuclear energy supply, face long-term sustainability challenges due to finite reserves. In contrast, the ocean harbors an estimated 4.5 billion tons of uranium—over 1000 times the terrestrial supply—representing a vast, untapped resource to secure millennium-scale nuclear energy development [82]. Addressing this urgent need for sustainable uranium sourcing, recent research designed a photoactive hydrogel made of carboxyl-functionalized g-C_3_N_4_/CdS (CCN/CdS) for uranium extraction [83]. The carboxyl groups on g-C_3_N_4_ enhance its affinity for uranyl ions, while cadmium sulfide promotes the activation of dissolved oxygen. Under ambient atmospheric conditions, the prepared photoactive hydrogel catalyst achieved over 80% reduction of 0.1 mM U(VI) within 150 min without the assistance of any electron donors (Figure 10a). No O_2_^−^ or ·OH radicals were detected in the dark, while strong O_2_^−^ signals and weak ·OH signals were observed under light irradiation. Photoelectrons reducing O_2_^−^ radicals generated a small amount of ·OH (Figure 10b). After photocatalytic reaction, the sample darkened due to the formation of reduced uranium oxide precipitates on the surface (Figure 10c). TEM images (Figure 10d) revealed the deposition of numerous small particles around the anchored regions of CCN/CdS, with U(VI) photocatalytic reduction efficiency dependent on the CCN/CdS content. The fringe spacing of the product matched the interplanar distance of the (111) plane of uranium dioxide (00-041-1422-ICDD). As shown in Figure 10e, a reduced binding energy of U 4f indicated electron capture, and the mixed-valent uranium in the product was attributed to the formation of UO_2+x_ (x < 0.25). Uniform distribution of Cd, S, and U confirmed that U(VI) photocatalytic reduction occurred around CCN/CdS particles (Figure 10f), consistent with TEM observations.

### 4.2. Synergistic Interfacial Evaporation and Photocatalytic Pollutant Degradation

Solar-driven interfacial evaporation (SIE) technology converts solar energy into thermal energy [84]. Unlike traditional bulk heating methods, thermal energy in SIE is confined to the evaporator surface, enabling seawater evaporation for freshwater production. This minimizes heat dissipation and enhances photothermal conversion efficiency. Recognized for its environmentally friendly, energy-saving, and renewable characteristics, SIE is regarded as a promising desalination technology [85]. Gel-based materials show great potential for application in SIE technology owing to their excellent water retention capability and thermal management properties. Photocatalytic technology utilizes photogenerated electron–hole pairs to carry out redox reactions, thereby degrading organic pollutants. Integrating solar-driven interfacial water evaporation with photocatalysis offers a green, sustainable, and efficient approach for simultaneous desalination and purification of water [86].

**Figure 11:**
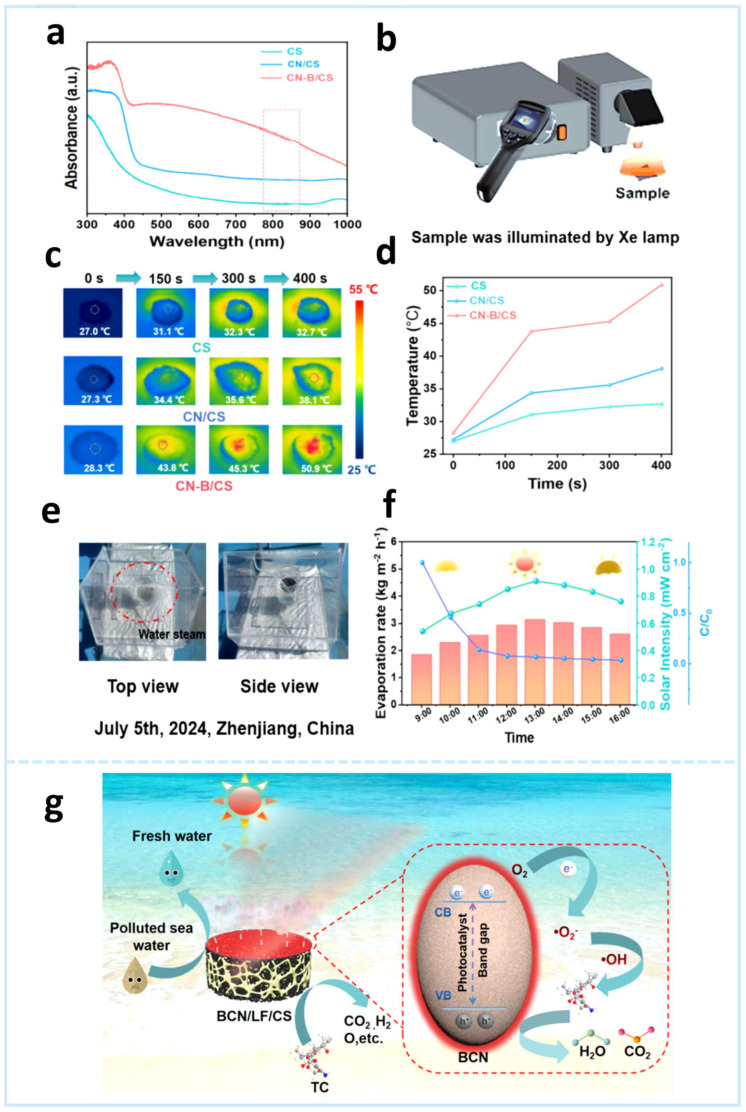
(**a**) UV–Vis-NIR absorption spectra of hydrogels (CS, CN/CS and CN-B/CS). (**b**) Schematic illustration of the experimental setup for photothermal performance evaluation. (**c**) Infrared thermography of CS, CN/CS, and CN-B/CS hydrogels under simulated 1-sun irradiation and (**d**) corresponding surface temperature distribution profiles of the hydrogels. (**e**) Photographs of outdoor solar desalination and organic pollutant degradation processes. (**f**) Outdoor experimental results of evaporation and pollutant degradation performances for CN-B/CS hydrogel [36]. (**g**) Conceptual schematic of synergistic desalination and TC degradation in the BCN-1/LF/CS solar evaporator [39].

CN-B (black g-C_3_N_4_), as a novel photocatalyst, possesses excellent light absorption properties and photocatalytic activity [87]. The introduction of the CN-B photocatalyst aims to enhance the photothermal conversion efficiency and photocatalytic degradation performance of the composite hydrogel. Chen et al. [36] prepared CN-B/CS composite hydrogels by mixing CN-B and chitosan (CS). The CN-B/CS hydrogel achieved an interfacial evaporation rate of 3.43 kg m^−2^ h^−1^ in a 3.5 wt% NaCl solution. Under 4 h of solar irradiation, it could achieve 82.5% degradation of tetracycline (TC) solution (100 mg L^−1^) while demonstrating excellent stability. Figure 11a shows limited optical absorption for CS, significantly enhanced in CN/CS within the visible range after CN incorporation. CN-B introduction further extended absorption into the NIR region due to its unique optical properties (Figure 11b). Infrared thermography (Figure 11c) revealed distinct photothermal responses under simulated sunlight: CS and CN/CS hydrogels reached stable temperatures of 32.7 °C and 38.1 °C after 400 s, while CN-B/CS surged to 50.9 °C due to the photothermal conversion of CN-B. Temperature rise profiles (Figure 11d) confirm the superior photothermal effect of CN-B/CS, enabling efficient solar-driven evaporation with photocatalytic degradation through light-to-heat conversion. Figure 11e shows water droplets on the glass cover from CN-B/CS hydrogel evaporation under solar illumination. Concurrently, TC solution concentration decreased (9:00–13:00) while evaporation efficiency increased significantly (Figure 11f).

Leveraging the exceptional mechanical properties, hydrophilicity, and 3D network structures of loofah sponge (LF) and CS to enhance evaporator stability and water transport efficiency, a novel multifunctional black g-C_3_N_4_/loofah sponge/chitosan (BCN/LF/CS) hydrogel solar interfacial evaporator was designed [39]. In this system, BCN efficiently converts solar energy into thermal energy for steam generation while generating abundant photogenerated electrons to degrade organic pollutants. Concurrently, LF and CS function as the structural backbone to enhance stability, facilitate water transport, and improve salt resistance. The optimized BCN-1/LF/CS evaporator achieved an effective evaporation rate of 2.1 kg m^−2^ h^−1^ in simulated seawater while maintaining 91.4% TC degradation efficiency (50 mg L^−1^, 6 h). As displayed in Figure 11g, the BCN/LF/CS dual-function evaporator operates via solar-driven interfacial evaporation and photocatalytic degradation. The LF/CS framework enhances stability, water transfer, and salt tolerance through its porous structure. BCN, as a novel photothermal material, efficiently converts sunlight to thermal energy. The integrated evaporator exhibits superior light absorption, rapid water permeation, and exceptional photothermal conversion, enabling high-efficiency freshwater extraction. Simultaneously, localized interfacial heat promotes charge separation in the BCN photocatalyst. Under sunlight irradiation, photoexcited electrons in the BCN conduction band react with dissolved oxygen to form ·O_2_^−^, while the valence band holes generate ·OH. These reactive species (·O_2_^−^; ·OH; h^+^) mineralize tetracycline (TC) into harmless small molecules (CO_2_; H_2_O).

**Figure 12:**
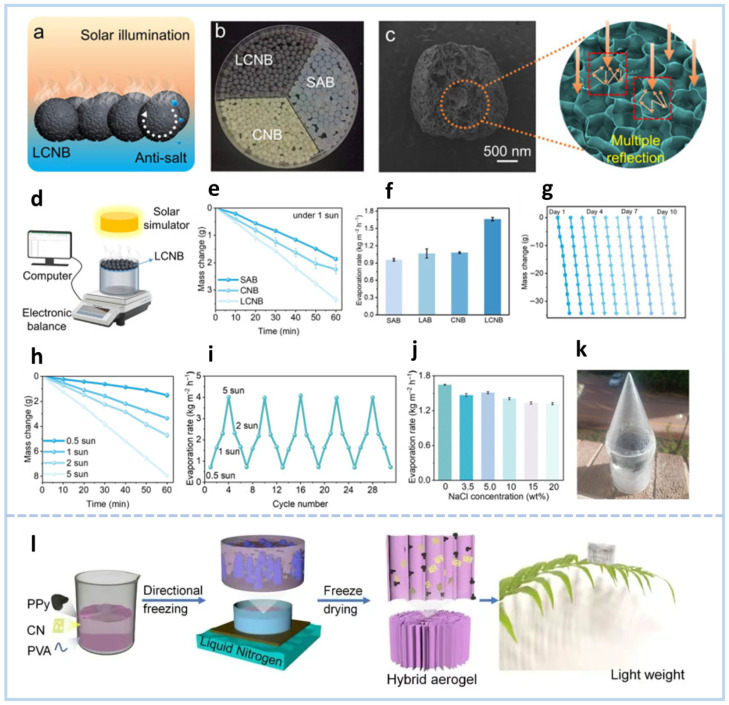
(**a**) Schematic illustration of LCNBs for continuous solar desalination. (**b**) Photographs of SABs, CNBs, and LCNBs. (**c**) SEM images of LCNB and its cross-sectional structure. (**d**) Schematic diagram of the solar evaporation device setup. (**e**) Water mass variation and (**f**) evaporation rates under 1-sun irradiation among different evaporators. (**g**) Long-term evaporation performance of the LCNB under continuous operation (10 h/day for 10 days, 1-sun illumination). (**h**) Evaporation rates of LCNBs to different solar irradiation intensities. (**i**) Cyclic stability of LCNBs under varying solar irradiation conditions. (**j**) Evaporation performance of LCNBs in NaCl solutions with varying concentrations. (**k**) Outdoor solar water collection device [88]. (**l**) Schematic illustration of the fabrication process for PCH-x aerogel/hydrogel [89].

Inspired by plant transpiration, a carbon nitride/polypyrrole/polyvinyl alcohol hydrogel (PCH) with vertically aligned channels was fabricated via directional freeze-drying for integrated interfacial photothermal and photocatalytic applications [88]. The self-floating LCNBs spontaneously assemble into a cohesive floating layer, concentrating heat at the evaporation interface to enable efficient solar-driven water evaporation (Figure 12a). As shown in Figure 12b, incorporating g-C_3_N_4_ into the composite beads (CNBs) imparts a distinct yellow hue compared to pristine white SA beads (SABs), while LCN addition yields a black coloration. LCNBs exhibit pronounced surface roughness with grooved textures and partial porosity, contrasting with the smoother surfaces of SABs and CNBs (Figure 12c). This textured morphology promotes multiple internal scattering events when light penetrates the material, effectively trapping incident radiation and enhancing light absorption capacity. Under a solar irradiance of 1 kW m^−2^, the LCNB evaporator achieved an evaporation rate of 1.64 kg m^−2^ h^−1^, significantly surpassing the performance of the SAB (0.97 kg m^−2^ h^−1^), LAB (1.12 kg m^−2^ h^−1^), and CNB (1.07 kg m^−2^ h^−1^) (Figure 12d–f). To evaluate the long-term durability of the evaporator, the LCNB device underwent 10 consecutive days of evaporation testing under 1-sun simulated illumination for 10 h each day. Notably, the evaporation rate remained nearly constant throughout the 10-day period, a stability directly attributed to its self-cleaning capability (Figure 12g). Furthermore, the evaporation rate exhibited a positive correlation with solar irradiance, reaching a peak of 4.01 kg m^−2^ h^−1^ under 5 kW m^−2^ (Figure 12h). Five consecutive outdoor simulation cycles further validated its robustness under fluctuating irradiance conditions (Figure 12i). Additionally, the seawater desalination performance of LCNB was tested with NaCl concentrations from 3.5 wt% to 20.0 wt%. Strikingly, the evaporation rates remained stable across these varying salinities, spanning 1.64 to 1.34 kg m^−2^ h^−1^ (Figure 12j). This indicates that LCNB can effectively operate in seawater, showcasing its potential for practical desalination applications (Figure 12k).

Rational design of lignin-derived carbon-doped g-C_3_N_4_ (LCN) enabled fabrication of spherical evaporators (LCNBs) via crosslinking with sodium alginate/PVA hydrophilic regulators and CaCl_2_ (Figure 12l) [89]. LCNBs self-assemble into dynamic planar systems achieving thermal localization, while gravity-induced rotation dissolves salt precipitates for continuous desalination. Carbon doping narrows bandgaps, enhancing photothermal/catalytic efficiency. Under 1-sun, LCNBs deliver 1.64 kg m^−2^ h^−1^ evaporation, zero salt accumulation in 20 wt% NaCl after 12 h, and 85.64% tetracycline degradation. These biomimetic prototypes provide a viable approach for dual mitigation of freshwater scarcity and water pollution, offering potential for scalable contaminated-water remediation and sustainable resource utilization [90].

## 5. Conclusions and Outlooks

### 5.1. Conclusions

g-C_3_N_4_ gels, encompassing hydrogels and aerogels, have emerged as a compelling class of materials for advanced water decontamination due to their tunable porosity, high surface area, and metal-free photocatalytic activity. This review has outlined the diverse synthesis strategies, from crosslinking and in situ polymerization to the sol–gel and template method, as well as post-synthetic modifications including chemical composition and structural engineering, all of which significantly enhance the physicochemical properties and environmental performance of g-C_3_N_4_ gels. These materials exhibit great promise in key applications such as photocatalytic degradation and solar-driven evaporation for desalination. However, challenges remain in scaling up production, ensuring long-term operational stability, and addressing the complexity of real-world water matrices. Continued interdisciplinary research focusing on structure function relationships, innovative fabrication routes, and practical implementation will be critical to fully realizing the potential of g-C_3_N_4_ gels in sustainable water purification technologies.

### 5.2. Outlooks

(1) Scalable and Sustainable Fabrication

Future efforts should prioritize energy-efficient, environmentally benign, and industrially scalable synthesis routes. Key strategies include simplifying multi-step procedures, advancing continuous production technologies (e.g., additive manufacturing/3D printing), and optimizing drying protocols (e.g., supercritical drying; freeze-drying) to enable commercial viability of g-C_3_N_4_ gels. Integration of green chemistry principles is essential [91]. For industrial-scale implementation, future efforts should prioritize scalable fabrication methods like continuous flow reactors for gel synthesis and roll-to-roll manufacturing for hydrogel film production, which could reduce costs by 30–40% compared to batch processes [92]. The inherent modularity of gel systems enables flexible deployment in existing water treatment infrastructures, from portable solar stills to industrial wastewater plants, with pilot studies demonstrating 90% cost recovery within 2–3 years of operation through reduced chemical and energy inputs [93].

(2) Enhanced Stability, Mechanistic Insight, and Real-Wastewater Validation

Improving long-term structural/chemical resilience under operationally harsh conditions (e.g., extreme pH; oxidizing environments) is imperative. This requires elucidating degradation pathways through advanced in situ/operando characterization techniques and rigorous performance validation in complex aqueous matrices, including real wastewater, to evaluate competitive solute effects, fouling behavior, and catalytic durability.

(3) Multifunctionality and Stimuli-Responsive Hybrid Systems

Synergistic integration of g-C_3_N_4_ gels with functional complements (e.g., MOFs, 2D nanomaterials, conductive polymers, and biomaterials) can yield multifunctional platforms for combined adsorption, (photo)catalysis, and (bio)sensing. Designing stimuli-responsive (“smart”) hybrids (e.g., pH-/temperature-/light-triggered) will expand their utility in adaptive, context-responsive environmental remediation [94].

(4) Life Cycle Sustainability and Circular Design

Comprehensive life cycle assessment (LCA) addressing cradle-to-grave environmental impacts, sustainable regeneration strategies (e.g., mild chemical washing; photoregeneration), and adoption of biodegradable/low-toxicity precursors are critical for ensuring the overall environmental footprint reduction. Emphasis on closed-loop material flows and end-of-life recyclability is paramount.

## Data Availability

No new data were created or analyzed in this study.
